# Improving Dispersion of Recycled Discontinuous Carbon Fibres to Increase Fibre Throughput in the HiPerDiF Process

**DOI:** 10.3390/ma13071544

**Published:** 2020-03-27

**Authors:** Thomas R. Pozegic, Samantha Huntley, Marco L. Longana, Suihua He, R. M. Indrachapa Bandara, Simon G. King, Ian Hamerton

**Affiliations:** 1 Bristol Composites Institute (ACCIS), School of Civil, Aerospace, and Mechanical Engineering, University of Bristol, Queen’s Building, University Walk, Bristol BS8 1TR, UK; tpozegic@yahoo.co.uk (T.R.P.); samantha.huntley@bristol.ac.uk (S.H.); m.l.longana@bristol.ac.uk (M.L.L.); silva.he@bristol.ac.uk (S.H.); 2 Advanced Technology Institute, Faculty of Engineering and Physical Sciences, University of Surrey, Guildford, Surrey GU2 7XH, UK; i.b.rajapakshemudiyanselage@surrey.ac.uk (R.M.I.B.); simon.g.king@surrey.ac.uk (S.G.K.)

**Keywords:** fibres, composites, discontinuous, surfactants, sustainable composites

## Abstract

In order to increase the material throughput of aligned discontinuous fibre composites using technologies such as HiPerDiF, stability of the carbon fibres in an aqueous solution needs to be achieved. Subsequently, a range of surfactants, typically employed to disperse carbon-based materials, have been assessed to determine the most appropriate for use in this regard. The optimum stability of the discontinuous fibres was observed when using the anionic surfactant, sodium dodecylbenzene sulphonate, which was superior to a range of other non-ionic and anionic surfactants, and single-fibre fragmentation demonstrated that the employment of sodium dodecylbenzene sulphonate did not affect the interfacial adhesion between fibres. Rheometry was used to complement the study, to understand the potential mechanisms of the improved stability of discontinuous fibres in aqueous suspension, and it led to the understanding that the increased viscosity was a significant factor. For the shear rates employed, fibre deformation was neither expected nor observed.

## 1. Introduction

Although for many years the preserve of aerospace, the use of advanced composite materials for engineering applications is becoming more widespread in an increasing variety of technological applications, including automotive, wind energy, marine, and civil engineering. However, as the use of composites becomes more mainstream, the issue of their sustainability becomes increasingly the subject of examination, and we have recently discussed this using the tool of life cycle assessment [1]. Fibre-reinforced polymer (FRP) composites that employ thermoplastic matrices have the potential to have the matrix recycled or reprocessed, but their use is still yet to gain traction in many applications, as the processing challenges associated with high melting temperatures, high melt viscosity, and high consolidation pressures are still problematic. In contrast, thermoset composites, e.g., epoxy-based, are more widely used, particularly in aerospace applications, but offer less opportunity for conventional recycling routes, since the crosslinked matrix is insoluble and infusible, and so requires extreme heat to char and remove it from the fibres. This in turn leads to complications with the carbon fibres (CFs), since during this recycling process the fibres are exposed to a degree of thermal degradation. This leads to the production of inherently ‘fluffy’ and highly entangled CFs post-reclamation. Consequently, recycled fibres are typically employed in non-structural applications—minimising the value of the reclaimed reinforcement [2].

Aligned Discontinuous Fibre Reinforced Composites (ADFRCs) have the potential to offer mechanical properties comparable with those of continuous fibre composites, provided that the fibre aspect ratio is high enough to allow load transfer and attain fibre failure instead of pull-out (i.e., fibre–matrix interface failure) [3]. The High Performance Discontinuous Fibre (HiPerDiF) technology, invented, patented, and developed at the University of Bristol [4], is an effective and sustainable high performance ADFRC manufacturing process, [5] for potentially high-throughput production [4]. ADFRCs produced using the HiPerDiF technique have been shown to overcome three key limitations of conventional continuous fibre composite materials: (1) their lack of ductility, addressed by hybridising different types of fibres (e.g., glass, carbon) or exploiting pull-out mechanisms [6,7,8]; (2) the difficulties in producing defect-free complex-shaped components as part of a high-volume automated manufacturing processes, and (3) the lack of a sustainable material lifecycle, e.g., the integration of production and end-of-life recycled waste in a circular economy model [2,9,10].

**Figure 1:**
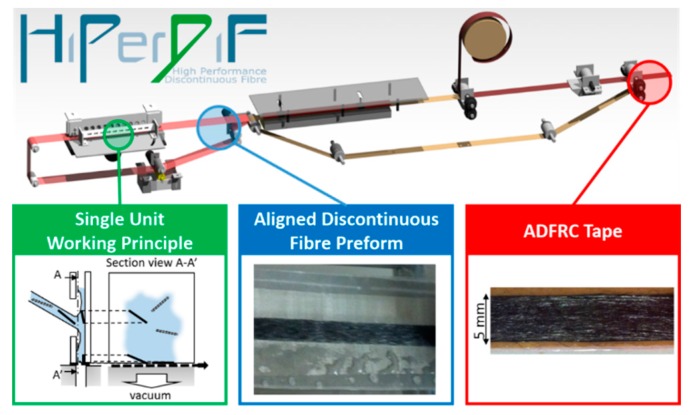
The HiPerDiF technology delivers aligned discontinuous fibres via a fibre-suspended water jet directed at an array of perpendicular plates. The removal of water leads to a highly aligned preform, to be later incorporated with a polymer matrix.

Within an EPSRC-funded project [5], we are currently scaling up the laboratory scale, second generation HiPerDiF discontinuous fibre alignment machine to produce a highly instrumented, high-throughput third generation machine, HiPerDiF 3G, capable of producing kg/hour quantities of discontinuous fibre prepreg. The HiPerDiF process (a schematic is shown in Figure 1) consists of discontinuous CFs suspended in water, which are accelerated through a nozzle, and directed to an array of parallel plates. The alignment mechanism relies on the momentum change of the fibres at the impact with the furthermost plate and the plate separation. The fibres deposit under gravity on to a stainless-steel conveyor mesh belt, where the majority of the water is removed by vacuum suction. The aligned fibre preform is transported along the conveyor belt and dried with infrared radiation. The dry, aligned fibre preform is subsequently coupled with a thermoset or thermoplastic matrix and partially impregnated through the application of heat and pressure. Different types and lengths of fibres can be blended in the water suspension, allowing intermingled [11], intraply [12], and interlaminated [13,14] hybrid FRP composites to be obtained. 

To realise the capabilities of ADFRCs via HiPerDiF methodology in the HiPerDiF 3G, further development is needed to assist with the scale-up of the technology. The current limitation of the second generation HiPerDiF machine is the maximum fibre content in the water medium prior to nozzle ejection. To enhance the maximum fibre volume fraction in the liquid phase, two aspects need to be addressed: the dispersion of agglomerates and the prevention of reagglomeration by stabilising the fibre suspension [15]. 

The dispersion of agglomerates is typically achieved through mechanical means (e.g., sonication, three roll mill, high pressure homogeniser, ball miller, or shear mixer [16]). However, particular care should be taken to ensure such mechanical methods do not degrade the physical properties. For instance, ultrasonication has been shown to result in a significant increase in the intensity of the D band (representing disordered *sp^3^* carbon bonding on carbon nanotubes) in Raman spectra [16]. This particular methodology is common in the dispersion of nanomaterials in a liquid phase [17], where adequate dispersion is a significant hindrance in fully exploiting the materials; as a result, there exists a significant amount of research in this area [16].

The stabilisation of the solid phase (fibres in this instance) in the liquid phase is attributed to the *zeta* potential, which is established on a surface when in contact with a liquid phase. Charge formation occurs due to reactions of functional groups or adsorption of ions from the liquid phase, co-ions are depleted from the surface, and counterions become sorbed at the surface. The potential at the surface decreases linearly to a value (at the centre of the adsorbed counter ions), and then decreases exponentially, reaching zero in bulk solution where the concentrations of the two opposite ion charges attain a constant average value [18,19]—typically in the order of 5–200 nm away from the surface [20]. Close to the surface, immobile charges are present (the Stern layer), beyond which, the shear layer defines the point at which ions will not move with the charged particle (the diffuse layer). The potential difference between the Stern layer and the diffuse layer to the surrounding suspending liquid is called the *zeta* potential, the magnitude of which can be enhanced through covalent or non-covalent functionalisation. 

Covalent functionalisation of CFs, of which there are many methods [16], is based on the covalent linkage of functional moieties on to the surfaces of the fibres, which is primarily formed of inert aromatic bonds (mixture of *sp*^2^ and *sp*^3^ hybridisation). Acids can be used to create defects for covalent functionalisation. Typically, CFs are treated through an oxidative process as part of an anodic electrolytic surface treatment, which results in oxygen functionalisation at the edges of the basal planes roughening the surface of the CFs, encouraging mechanical interlocking [21]. The treatment and covalent bonding between the polar groups on the surface and the polymer matrix removes the weak surface layer on the fibre, allowing the matrix to bond to a stronger layer underneath [22]. This functionalisation can incur damage (i.e., acid treatments) and can alter the *sp*^2^ bonds which provide the principal mechanical performances.

Non-covalent functionalisation involves a variety of methods such as using a surfactant or a co-polymer [23,24]. In the case of the latter, the lyophobic part of the block co-polymer positions itself close to the fibre to reduce the overall free energy, while the lyophilic block positions itself towards to the solution. When surfactants are employed, the physical adsorption of a surfactant lowers the surface tension of the fibre and prevents agglomeration; this is caused by the hydrophobic attraction between the fibre and the surfactants tail group, whilst the hydrophilic segment interacts with the liquid phase through hydrogen bonding. Surfactant molecules self-assemble into micelles when the concentration exceeds the critical micelle concentration (CMC). The level of adsorption of surfactant on the fibres in water depends on a number of factors, including the nature of the structural groups on the solid surface, the molecular structure of the surfactant, and the condition of the aqueous phase, such as pH and temperature [25].

Another material used in composites which suffers similar solubility issues as CFs are carbon nanotubes (CNTs), which are typically nano-width sized graphitic carbon tubes composed of mainly *sp^2^* and some *sp^3^* carbon bonds, thus significant work has been conducted on optimising dispersions of them [26,27]. As such, similar methodologies can also be applied to the CFs used in this study. Gong et al. [28] utilised surfactant-assisted processing of CNT composites with a non-ionic surfactant, which led to improved dispersion and interfacial bonding of the CNTs in an epoxy matrix, resulting in a 30% increase in the elastic modulus with the addition of only 1 wt% of CNTs. For non-ionic surfactants a different adsorption mechanism occurs, as there is no charge attraction; adsorption is driven purely by hydrophobicity. Longer hydrocarbon tails will be preferred, and the presence of an unsaturated C=C bond will be advantageous as a result of the π-electronic affinity of the surfactant towards CNT benzene rings. On the other hand, the hydrophilic chain will promote surfactant solubility in the aqueous phase, reducing the adsorption ratio on to the nanotubes [29].

Vaisman et al. [27] state that ionic surfactants are preferable for CNT–water soluble solutions, whilst non-ionic surfactants are preferred in organic solvents. Furthermore, it has been established that there is no preference for anionic or cationic species. Among the ionic surfactants, sodium dodecyl sulphate (SDS) and sodium dodecylbenzene sulphonate (SDBS) have been commonly used to decrease the tendency of CNTs to aggregate in water [30]. The benzene ring of the surfactant was proposed to be responsible for the high dispersive efficiency of SDBS with the π-stacking interactions of the benzene rings on the CNT surface believed to increase the adsorption ratio of surfactants [31], as well as of other highly aromatic molecules [32], and rigid conjugated polymers [33]. Vasiman et al. [29] concluded that surfactants which have long tail groups and unsaturated C=C bonds contribute greatly to the stabilisation of the CNTs. 

The application of surfactants for processing of CFs is limited: Giraud et al. [34] utilised SDS and benzalkonium chloride in applying a thermoplastic sizing to unsized polyacrylonitrile (PAN) CFs and determined that SDS successfully provided a homogenous film of sizing. Yang et al. [35] compared a range of surfactants to disperse discontinuous CFs and reported that the non-ionic surfactant Triton™ X-100 provided the best dispersion, partly driven by the hydrophobic interactions between the aliphatic carbon chains in PAN CFs and the hydrophobic tails of Triton™ X-100 [36]. Chuang et al. [37] dispersed PAN CFs using different derivatives of cellulose and reported that the hydroxyethyl cellulose was the best dispersant of those tested—the greater abundance of hydroxyl polar groups to form hydrogen bonds or to bridge with the polar groups on the surface of the CF improved the dispersion. 

The target of this current work is to determine an ideal solution for increased throughput and methods to quantify the enhancements (i.e., dispersion) and to gain insight into the stabilising mechanism. Furthermore, mechanical testing at the single fibre level will quantify any changes to the fibre–matrix interfacial interactions. 

## 2. Methodology

### 2.1. Materials

Granoc XN-90 (NGF), a pitch-based fibre, was used throughout this study; the low strain to failure rate is ideal for single fibre fragmentation testing and, furthermore, the fibre is commonly used with the HiPerDiF programme as a high modulus carbon fibre (hereinafter named ‘HMC’). The polymer matrix used for the single fibre fragmentation test was Gurit Prime^TM^ 27 with a slow polyamine hardener (Gurit). The properties obtained from the respective manufacturer of both fibre and matrix can be found in Table 1, respectively. For the fibre fragmentation tests, the polymer matrix was cured as per the manufacturer’s guidelines: 7 hours at 65 °C. The following surfactants were all purchased from Sigma-Aldrich (Merck, Poole, UK) and used as received: poly(vinyl alcohol) (PVA) (average M_w_: 146,000–186,000 g/mol.; 87–89% hydrolysed), Brij^®^ 58, Pluronic F127, sodium dodecyl sulphate (SDS), sodium dodecylbenzene sulphonate (SDBS), Triton™ X-100, Triton™ X-405, and Tween^®^ 80. For the dissolution experiments, the HMC fibres were chopped into 3 mm lengths.

### 2.2. Fibre Characterisation 

#### 2.2.1. Thermal Stability

To aid reproducibility and clarity in the task of modifying and analysing the fibre surface, the sizing was removed by heating the fibres to 500 °C for 3 hours in air. The thermal stability of the fibres was assessed to determine the temperature to remove the polymer sizing whilst minimising damage to the fibres. The polymer sizing is typically a ~1 wt% epoxy resin varnish applied to the fibre to aid handling and processing [38]. To study the thermal stability, a simultaneous thermal analysis (STA) instrument (Netzsch STA 449 F1 Jupiter, Selb, Germany) was used; this allows for thermogravimetric analysis (TGA) and differential scanning calorimetry (DSC) data to be obtained. The fibres (ca. 10 mg) were added to Al_2_O_3_ crucibles and subjected to a temperature programme from 30 °C to 600 °C at 10 K/min, under air atmosphere (50 cm^3^/min).

#### 2.2.2. *Zeta* Potential

The surface charge of the HMC fibres was determined using a streaming *zeta* potential analyser (SurPASS 3 Electrokinetic Analyzer, Anton Paar, St. Albans, UK). A liquid phase is forced under a pressure gradient, accumulating electric charges down-stream, causing a build-up of an electric field which drives an electric current back (by ionic conduction through the liquid) against the direction of the liquid flow. A steady state is established and the measured potential difference across the capillary or plug is called the streaming potential, this is directly related to the driving pressure [20]. The *zeta* potential and the isoelectric point (IEP) were determined using a cylindrical cell in the instrument, in the presence of aqueous samples of KCl (1 × 10^−3^ M) at various pH values, adjusted with 0.05 M HCl or 0.05 M KOH. The cell was titrated in the direction of the IEP, then subsequently rinsed repeatedly with aqueous KCl (1 × 10^−3^ M) (3 × 200 ml and 2 × 200 ml). Thereafter, a pH range from ~3 to 9 was swept. The *zeta* (ξ) potential is calculated from the streaming potential measurements according to the following equation:

(1) $$\xi =\frac{dU}{dp}\frac{\eta }{\varepsilon \varepsilon_{0}}\kappa$$

where dU/dp is the streaming potential versus the differential potential pressure, *η* is the electrolyte viscosity, *ε_0_* is the permittivity and *ε* is the dielectric coefficient of the electrolyte, and κ is the bulk electrolyte conductivity. 

### 2.3. Dissolution

A range of surfactants typically employed for the dispersion of carbon-based particulates and CFs within a liquid phase, were initially assessed for their ability to stabilise the discontinuous HMC fibres [26] and are listed in Table 2. 

In the preliminary tests, concentrations of 1.1 mg/mL, 11 mg/mL, and 110 mg/mL of each surfactant were added along with HMC (22 mg) and deionised water (20 ml) in a centrifuge tube. The solution was subsequently tip sonicated (Cole Palmer CPX750—750 W max., Cole Palmer, St. Albans, UK) for 10 min at 40% power, with pulse 5 seconds on, 5 seconds off, and using a 13 mm tip probe. For all samples, the tip was positioned at the same location (~20% into the solution) with respect to the vessel/solution to minimise the dispersion variation from the processing technique. King et al. [26] assessed the efficiency of sonication, varying sonication power, and horn depth, and imaging the sono-intensity within the solution using luminol, they found that the optimum horn depth was best placed at 20% into the solution volume.

Following the preliminary test, aqueous solutions of SDBS were prepared in concentrations of 13.92, 34.80, 69.60, 104.40, and 139.20 mg/mL with deionised water (30 ml). Foam was removed using isopropanol alcohol. HMC fibres with the sizing removed (ca. 33 mg), were added to the solution and tip sonicated (Branson 450, Cole Palmer, St. Albans, UK) for 3 min, 2 seconds on, 5 seconds off at 20% power using a narrow tip. Solutions were magnetically stirred for 5 min and photographs of the sample were taken at t = 0, 1, 2, 3, 4, and 5 min, denoting the time elapsed after the stirring ceased. Solutions were agitated with a magnetic stirrer for ~20 seconds and added to a centrifuge tube and centrifuged for 30 min at 7500 G. The supernatant was removed, and the dropped-out material was weighed after drying in the vacuum oven, providing a measurement of the mass of CFs which remained dispersed. This technique was successfully employed by King et al. [26] for CNTs. Hereinafter, the different concentrations shall be reported as stated in Table 3.

### 2.4. Rheology

A TA Discovery HR-1 (TA Instruments, Elstree, UK) with a 40 mm/4° stainless-steel cone-plate geometry (97 µm gap), and a Peltier plate was used to determine the viscosity of the solutions, which was recorded as a function of shear rate between 1 and 150 s^−1^.

### 2.5. Single Fibre Fragmentation

**Figure 2:**
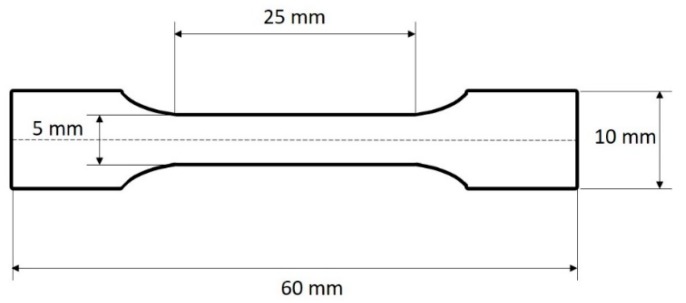
Schematic diagram of dog-bone shaped sample used for single fibre fragmentation tests.

Continuous HMC fibres (with sizing removed) were aligned in dog-bone shaped moulds (Figure 2) and kept under tension using tape on both ends. For the surfactant-treated samples, the mould was used as a bath to submerge the fibres in the SDBS_5 solution. After 12 hours, the solution was removed, and the fibres were left to dry for 1 hour. Prime^TM^ 27 was prepared as per the manufacturer’s directions and added to the moulds. The epoxy resin was cured at 65 °C for 7 hours. 

Samples were tested using a Shimadzu AGS-X tensile tester (Milton Keynes, UK) fitted with 10 kN load cell. Tests were conducted at a constant crosshead speed of 0.2 mm/min and elongated by 2 mm [39], satisfying the criterion that the matrix is required to have a strain to failure three times greater than that of the fibre [39]. Samples were analysed using a Zeiss Imager M2 optical microscope (Carl Zeiss Vision, Birmingham, UK). The interfacial shear strength, τ, was calculated with the following equation:

(2) $$\tau =\frac{\sigma_{f}\left(l_{c}\right)d_{f}}{2l_{c}}$$

where *σ_f_(l_c_)* is the fibre strength at the critical length, *d_f_* is the fibre diameter, and *l_c_* is the critical fragment length of the fibre ($l_{c}=4l/3$) [40], determined by measuring fractured fibre length using an optical microscope. 

## 3. Results

### 3.1. Fibre Characteristics

**Figure 3:**
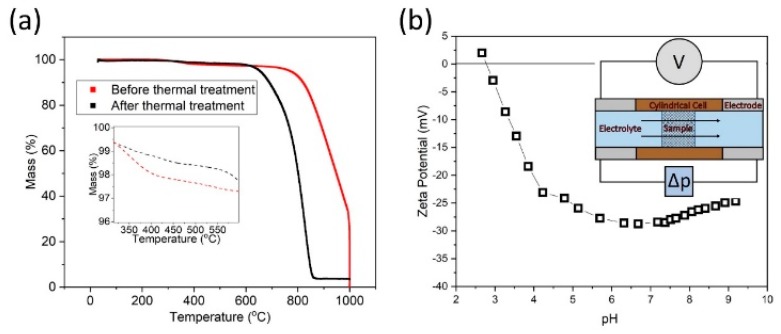
Characteristics of the HMC fibre. (**a**) The thermogravimetric response of the HMC fibres, (inset): in the region of the degradation point of the polymer sizing. (**b**) The streaming *zeta* potential of HMC fibres. (For interpretation of the references to colour in this figure, the reader is referred to the web version of this article).

To confirm that the polymer sizing had been removed successfully, TGA was conducted on the fibres following the thermal treatment (Figure 3, black curve). The results demonstrate successful removal, as typically, the response of a sized fibre (Figure 3, red curve) would be evident by a mass drop between 200–400 °C [41], attributed to the volatilisation and thermal degradation of sizing agent, which is typically a low molecular weight epoxy resin. It should be noted that the thermal treatment has reduced the onset of the critical-strength loss temperature by some 150–170 °C, suggesting potential damage to the fibres.

The streaming *zeta* potential of the sized-removed HMC, as seen in Figure 3b reveals an isoelectric point (IEP) of ~2.8 pH and suggests stability from pH ~4 to 9. The isoelectric point (IEP) is the pH where the *zeta* potential is zero and where the colloidal system is least stable. Subsequently, net negatively- and positively-charged surfaces can be achieved by pH control of the solution [42], by influencing the dissociation of surface groups. Dissociation of acidic groups will lead to negatively charged surfaces, whilst basic groups will lead to positively-charged surfaces, although the utility of a positively-charged HMC fibre surface is somewhat limited in this context. The magnitude of the *zeta* potential dictates the stability of the fibre solution: high *zeta* potentials (i.e., greater than ± 30 mV to ± 60 mV) are electrically stable particles with a *zeta* potential magnitude greater than ±15 mV are expected to be stable from electrostatic considerations [43], but particles with *zeta* potentials between -15 and 15 mV can still be stable if they are stabilised sterically [30]. Steric hindrance through the addition of polymers can be impractical and expensive, leading to additional complications if they need to be removed to maintain purity.

### 3.2. Dissolution

Through comparing a range of surfactants, typically used in dispersing carbon-based materials [26], the surfactant that led to the most stable suspension was SDBS; after 5 min, fibres remained suspended in the solution. The only other surfactant which gave stable fibre suspension at t = 5 min was Pluronic F127. Interestingly, in terms of their CMC, Triton™ X-100, Brij^®^ 58, and Tween^®^ 80 are all significantly higher, but this did not lead to improved fibre stability in solution, suggesting either poorer adsorption of the surfactant on the surface, or that the resulting viscosity was less. However, it is not known to what extent the CMC has shifted with the incorporation of the HMC fibres for Triton™ X-100, Brij^®^ 58, and Tween^®^ 80.

**Figure 4:**
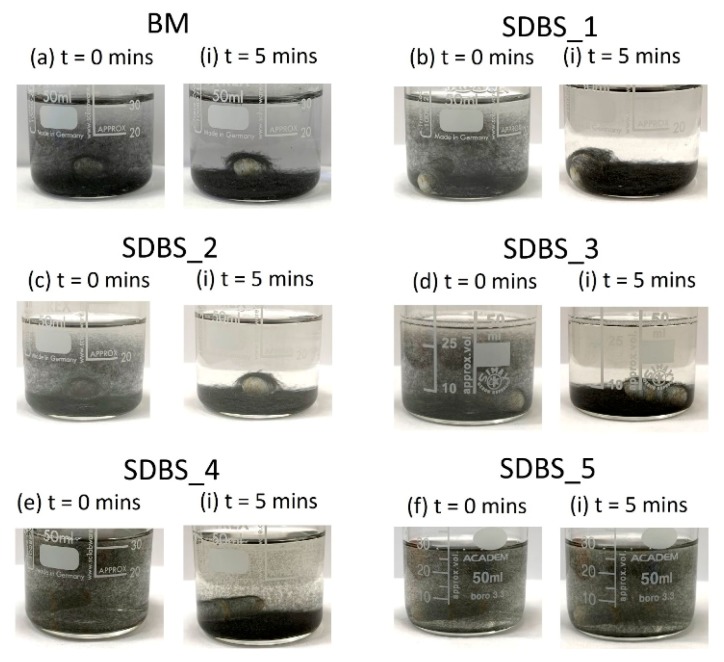
Dissolution experiment at t = 0 and t = 5 min for different concentrations of SDBS (**b–f**), compared to the (**a**) benchmark sample (BM). Shorter fibres are suspended in SDBS_4, whereas the majority of fibre lengths are suspended in SDBS_5.

The results of the preliminary test informed the main dissolution experiment, both for surfactant type and concentration. After tip sonication and agitation using a magnetic stirrer, the fibre-SDBS solution was left in a steady state and photographed every minute for 5 minutes. The results can be seen in Figure 4 for t = 0 and t = 5 min, for each concentration and a benchmark sample (no surfactant). Evidently, fibres display improved stability when the surfactant solution is >104.4 mg/mL (SDBS_4 and SDBS_5). The number of fibres per unit volume leads to a semi-concentrated regime, nL^3^ ~ 57 (nL^3^ << (L/d)^2^) [44], however, upon agitation (i.e., stirring), the fibres are less likely to be uniformly distributed, leading to areas of higher/lower concentration.

The solutions were agitated using magnetic stirring and were added to a centrifuge for 30 min at 7500 G. These conditions have been reported to result in compressed, precipitated material that could be weighed, and thus, used to determine dispersal power [26]. However, for discontinuous fibres, the phase between precipitated material and supernatant was not clear, making comparisons between each centrifuged sample difficult. Furthermore, it was noted for SDBS_5 that surfactant residue was visible in the precipitated material, essentially hindering measurement accuracy. 

SDBS is a widely used ionic surfactant, the benzene ring can form π-π conjugate with the *sp*^2^ hybrid bonds on the surface and the polar sulphonate is helpful to disperse in polar water [45]. The interaction of the anionic SDBS will be partly driven by the hydrophobic interaction between the tail groups, the fibre surface, and the repulsion of the negatively-charged head groups [29]. Gallardo-Moreno et al. [46] and Gupta Bhagwat et al. [47] reported strong adsorption of anionic SDS on parts of a carbon black surface, adding that the electrostatic repulsion between surfactant and particles was overcome by specific adsorption of the surfactant at the natural pH of the solutions [48]. Sis et al. [48] observed a reduction in the absolute magnitude of the *zeta* potential, and concluded that the addition of the non-ionic surfactant shifted the shear plane position away from the carbon surface as a result of the formation of a polymer layer due to adsorption [42]. Furthermore, they observed a substantial increase across a wide pH range employing anionic surfactant sodium oleate with carbon black. 

**Figure 5:**
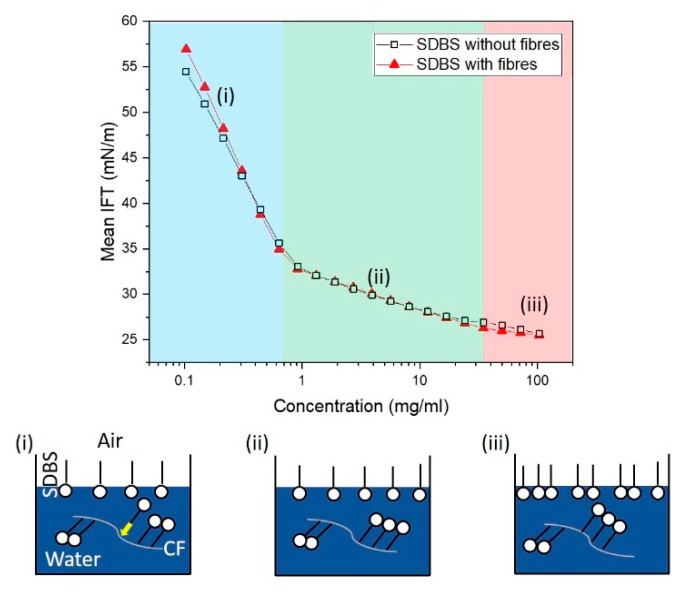
The reduction in the surface tension of the aqueous–air surface with the concentration of SDBS surfactant. The hypothesised adsorption of the SDBS to the fibre is proposed (**i–iii**), where early saturation of the CF is proposed relative to the concentrations tested. (For interpretation of the references to colour in this figure, the reader is referred to the web version of this article).

The concentrations tested reside where the surface tension (between the aqueous solution and air) plateaus, as shown from the tensiometer result (Figure 5). To understand potential sorption of surfactant to CF, the same solution was prepared with and without CFs. Prior to the plateau (Figure 5, phase iii) there are two distinct phases (Figure 5i, ii). The initial phase (Figure 5i) from SDBS concentrations 0.1 mg/mL to 0.6 mg/mL results in the greatest reduction in surface tension, leading to the vicinity of the CMC point (~1 mg/mL [49]). The majority of the SDBS assembles at the air/liquid interface during this phase. Between 1 and 35 mg/mL (ii), there is a reduction in gradient suggesting the SDBS molecules are not forming on the air/liquid interface as readily as (i). Beyond 35 mg/mL, there is a further distinct phase in the reduction of the gradient to an approximate plateau. For solutions with and without fibres, there is a difference in phase i, suggesting possible assembly at the CF interface inhibiting the reduction in surface tension at the aqueous solution–air interface. However, this difference diminishes at higher concentration (phase ii), leading to a small, possibly insignificant difference in phase iii. This result could potentially lead to the hypothesis that: there is migration of the SDBS molecule and assembly leading to early saturation on the CF at relatively low concentrations (see hypothesis in Figure 5); the tensiometer is insensitive to differences between with and without CFs, or the SDBS molecule absorbs poorly on the surface of the CF.

### 3.3. Single Fibre Fragmentation

**Figure 6:**
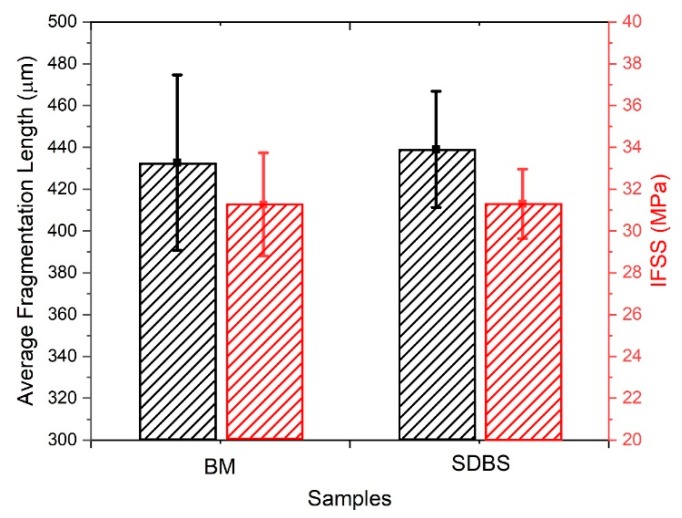
Average fragment length and the interfacial shear strength (IFSS) for benchmark samples (BM) and the SDBS modified samples. (For interpretation of the references to colour in this figure, the reader is referred to the web version of this article).

Given the potential modification to the surface of the HMC and the effect on the final composite product, performing a mechanical characterisation of the fibre–matrix interface is crucial. Both unmodified and modified samples underwent identical thermal treatments, hence the variable of tensile properties is removed. The single fibre fragmentation test was performed on sized-removed, benchmark samples (n = 5) and surfactant-coated HMC fibres (n = 5). The fragmented lengths for each fibre type were measured to be within error (of the mean) for both BM and surfactant modified samples: (433 ± 42) µm for BM and (438 ± 6) µm for the SDBS modified sample, as can be seen in Figure 6. Outliers were determined using boxplots (three box-lengths and greater), leading to the following numbers of data entries: BM (n = 22) and SDBS (n = 21). The interfacial shear strengths (IFSS) for BM and SDBS were normally distributed, as assessed by Shapiro-Wilk’s test (*p* > 0.5). There was homogeneity of variances for engagement scores for BM and SDBS, as assessed by Levene’s test for equality of variances (*p* = 149). Data are mean ± standard error in mean. Mean BM was (31.3 ± 2.5) MPa and for SDBS, (31.3 ± 1.7) MPa. An independent-samples t-test was performed to determine whether there were differences between the results of the single fibre fragmentation tests for BM and SDBS. The differences between the IFSS of BM and SDBS were not statistically significant—t(41) = 0.009, *p* = 993 (*p* > 0.5).

### 3.4. Viscosity

**Figure 7:**
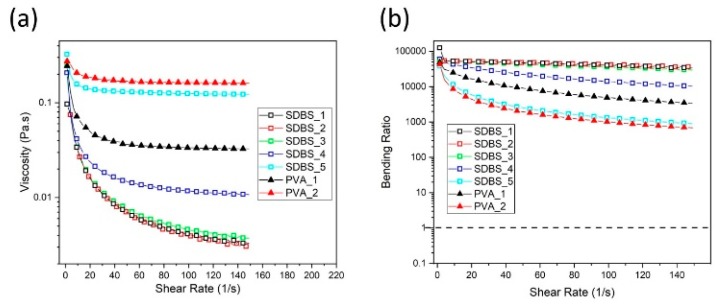
(**a**) The rheological response of the aqueous–SDBS solution. (**b**) The bending ratio with respect to shear rate for each of the aqueous solutions. The dashed line in (**b**) refers to the prediction of fibre bending. (For interpretation of the references to colour in this figure, the reader is referred to the web version of this article).

To account for the contribution of the viscosity of the added surfactant, rheometry was conducted on the different concentrations of aqueous SDBS solutions and the results can be observed in Figure 7a. The aqueous solutions demonstrate shear thinning behaviour, in particular, for the solutions with the lowest SDBS concentration. Evidentially, when the concentration increases from 69.6 mg/mL (SDBS_3) to 104.40 mg/mL (SDBS_4) and 139.20 mg/mL (SDBS_5), the viscosity notably increases. This coincides with the dispersion performance of these two high surfactant solutions (104.40 mg/mL and 139.20 mg/mL; SDBS_4 and SDBS_5, respectively). 

To determine the possibility of the viscosity being a mechanism to enhance fibre stability, aqueous PVA solutions with rheological responses within the vicinity of SDBS_4 and SDBS_5 were tested to determine the stability of the discontinuous fibres, as per Section 3.2. The rheological responses of PVA_1 (37.21 mg/mL) and PVA_2 (55.17 mg/mL) can be viewed in Figure 7a. PVA_1 resides between SDBS_4 and SDBS_5, with PVA_2 within the vicinity of SDBS_5. As the shear rate approaches 0, SDBS_4, SDBS_5, PVA_1 and PVA_2 converge. Consequently, the contribution of the viscosity during the dissolution experiment can be assessed, as the shear rate reduces to 0 as the magnetic stirrer is turned off.

**Figure 8:**
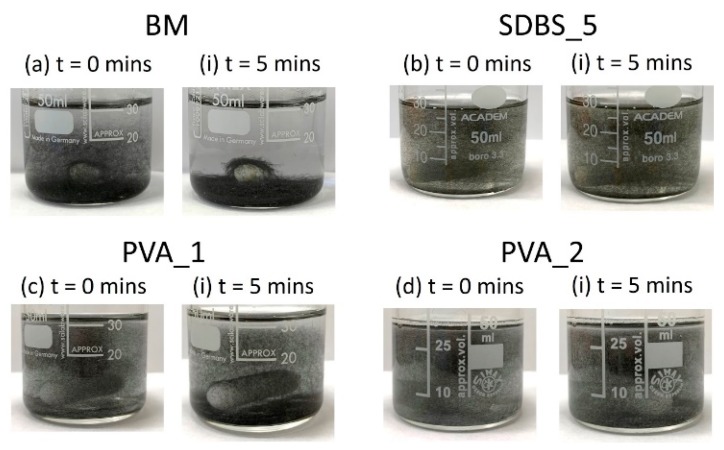
Dissolution experiment at t = 0 and t = 5 min for (**a**) BM (**b**) SDBS_5, compared to (**c**) PVA_1 and (**d**) PVA_2.

The aqueous PVA solutions were tested to evaluate their ability to stabilise the sized-removed discontinuous HMC. Images were taken at the moment agitation (magnetic stirring bar) was turned off and 5 minutes later (Figure 8). As a comparison, BM (a) and SDBS_5 (b - best stability) are included as a comparison with PVA_1 (c) and PVA_2 (d). Evidently, all solutions display strong stabilisation with minimal settling. Furthermore, PVA_1 and PVA_2 look to be comparable to SDBS_5. This would suggest that viscosity is the main mechanism for stability for the SDBS aqueous solutions. 

It is well understood that viscosity (*η*) is inversely proportional to the settling velocity (*V*) and can therefore slow settling speed, as described by Stoke’s law:

(3) $$V=\frac{2\Delta \rho ga^{2}}{9\eta }$$

where Δρ is the density difference between settling sphere (of radius, *a*) and liquid and *g* is the acceleration due to gravity. However, if the viscosity is too high, this can lead to fibre bending. Forgacs et al. [50] and Goldsmith et al. [51] investigated the induced deformation of single fibres in simple shear flow:

(4) $$BR=\frac{E_{x}\left(\frac{2.48\left(L/d\right)}{{\left(\ln\left(L/d\right)\right)}^{0.5}}-1.50\right)}{2\eta \dot{\gamma }{\left(L/d\right)}^{4}}$$

where *BR* is the bending ratio, *E_X_* is the elastic modulus, *L/d* is the fibre aspect ratio, $\dot{\gamma }$ is the shear rate. Fibre bending is predicted when *BR* < 1. Subsequently, BR was determined and plotted against shear rate in Figure 7b. It can be observed that for all aqueous solutions, the criterion of preventing induced bending deformation (BR > 1) is met (dashed line in Figure 7b), concluding that fibre bending is not expected, which is in agreement to our observations during the dissolution experiments. Fibre bending would be detrimental to discontinuous fibre alignment technologies, seeking to maximise the value in reclaimed fibres, as such, an upper limit of viscosity should be adhered to prevent fibre deformation.

## 4. Conclusions

Stability of discontinuous fibres was observed using the surfactant sodium dodecylbenzene sulphonate which was superior to other non-ionic and anionic surfactants. The addition of the surfactant does not have an effect on the interfacial adhesion, as demonstrated by the single fibre fragmentation data. The dominant mechanism to stabilise the fibres was the increase in solution viscosity and was demonstrated by testing a similarly viscous poly(vinyl alcohol) aqueous solution and comparing the fibre settling times. This result could be a consequence of the poor adsorption of the SDBS to the CFs, as evidenced by the tensiometer experiment. As viscous fluids are capable of bending the carbon fibres, which would be detrimental to technologies such as HiPerDiF which align and maintain mechanical performance of the fibres, the possibility of bending was predicted as not likely and reinforces our observations made during the experiments. To conclude, increasing the viscosity is an effective way to stabilise the CFs and can be achieved without deforming the fibres. Future work could focus on achieving effective adsorption of surfactant molecules of CFs to minimise the need to increase surfactant concentration, andviscosity which could be detrimental for processing (mechanical stirring or drying fibres).

## Figures and Tables

**Table 1:** Fibre and polymer matrix properties.

Fibre Property	High Modulus Carbon Fibre	Polymer Matrix
Commercial Name	Granoc XN-90	PRIME^(TM)^ 27
Density (g/cm^3^)	2.21	1.139 (Cured)
Diameter (µm)	10	–
Young’s modulus (GPa)	860	3.47
Tensile strength (MPa)	3430	73.3
Failure Strain (%)	0.398	4.5

**Table 2:** List of surfactants used. List reproduced from: [26].

Surfactant	Chemical Name	Molecular Weight (g/mol.)	Surfactant Type
Brij^®^ 58	Poly(ethylene glycol) hexadecyl ether	1124	Non-ionic
Pluronic F127	Poloxamer 407	12500	Non-ionic
SDS	sodium dodecyl sulphate	288	Anionic
SDBS	sodium dodecylbenzene sulphonate	348	Anionic
Triton™ X-100	Poly(ethylene glycol) *tert*-octylphenyl ether	625	Non-ionic
Triton™ X-405	Poly(ethylene glycol) *tert*-octylphenyl ether	1968	Non-ionic
Tween^®^ 80	Poly(ethylene glycol) sorbitan monooleate	1310	Non-ionic

**Table 3:** Identifier for different concentrations.

Concentration (mg/mL)	Identifier
13.92	SDBS_1
34.80	SDBS_2
69.60	SDBS_3
104.40	SDBS_4
139.20	SDBS_5
